# Confronting the figure of the “mad scientist” in psychedelic history: LSD’s use as a correctional tool in the postwar period

**DOI:** 10.3389/fpsyg.2023.1129428

**Published:** 2023-07-13

**Authors:** Andrew Jones

**Affiliations:** Institute for the History and Philosophy of Science and Technology, University of Toronto, Toronto, ON, Canada

**Keywords:** psychedelic history, LSD, prisons, Canada, mad scientists

## Abstract

Since reports about CIA-funded LSD studies came out in the 1970s, psychedelic drugs have invoked images of unethical experimentation and “mad scientists” in the public imagination. Even now, as the stigma surrounding psychedelics diminishes in the 21st century, the figure of the “mad scientist” continues to occupy a space in what Ido Hartogsohn calls the “collective set and setting,” the larger framework of cultural understandings that shape how individuals experience psychedelic drugs. Scientists and humanities scholars who study these drugs have responded to this issue by drawing boundaries between those who used psychedelics carefully and those who used them ignorantly. Yet these boundaries were not always so clear in the past. Drawing on historical examples of LSD’s use as a *correctional tool* in Canada, I show how enthusiasm about the drug’s potential led several experienced and knowledgeable psychedelic therapists to use it on vulnerable populations in diverse institutional settings, such as correctional facilities. These examples reveal how the institutional context of modern industrial societies shaped the application of psychedelic therapy in the past and suggest that today’s therapists need to carefully consider how this broader context impacts their work.

## 1. Introduction

In 2001, The *Ottawa Citizen*, an influential Canadian newspaper, ran an article titled “LSD Experiments: How a Mad Scientist Went Unchecked.” The article described the recent controversy surrounding the history of experimentation in Canadian correctional facilities, which emerged after several former inmates from the Kingston Prison for Women accused the prison’s psychologist of giving them lysergic acid diethylamide (LSD) in the early 1960s. Investigation into these accusations revealed that the Chief Psychologist at the prison, Mark Eveson, began a pilot study in 1961 that explored whether the drug could help in the rehabilitation of 23 inmates. The article went on to compare Eveson’s use of LSD to the psychiatrist Ewen Cameron’s CIA-funded “psychic driving” experiments in Montreal, noting that Cameron was “the closest thing Canada has produced to a real-life mad scientist” (Bronskill and Blanchfield, 2001).

As this news article reveals, psychedelic drugs have been closely associated with “mad scientists” and unethical experimentation in the public imaginary. This association emerged in the late 1970s, when the American journalist John Marks wrote about a network of CIA-funded researchers who covertly explored LSD as a weapon and truth serum (Marks, 1979). Over the following decades, more stories of LSD experimentation on vulnerable populations made headlines, and by the end of the 20th century, psychedelics were not only closely tied to drug abuse, but also with abuse of subjects in a scientific context (Dyck, 2008, p. 4).

Even now, as cultural attitudes toward psychedelics are changing, the image of the mad scientist continues to occupy a space in what Ido Hartogsohn has called the “collective set and setting,” the larger framework of cultural understandings that shape how individuals experience these substances (Hartogsohn, 2020). In addition to drug laws, fashion trends, and economic attitudes, stories about abusive scientists and unethical experimentation influence how we view psychedelics. This is especially evident while working in the humanities, where presentations about the history of LSD are often met with questions such as, “What about MK-ULTRA and the CIA?”

As humanities scholars who study psychedelics, what should we do about this image of the mad scientist that continues to lurk in the background of our collective set and setting? Those at the forefront of scientific investigation into psychedelics have their own approach to this issue: boundary drawing. Sociologists and anthropologists of science have shown that today’s psychedelic scientists carefully distance themselves from the irresponsible or ethically dubious actions of past psychedelic icons, such as Timothy Leary (Corbin, 2012; Langlitz, 2013; Giffort, 2020). By doing so, current researchers maintain a respectable image and help bring psychedelic science into the mainstream. Humanities scholars often adopt a similar strategy. Historians, for example, have challenged the association between LSD and unethical research by drawing boundaries between pioneers who used the drug properly and researchers who used it naively. From this perspective, the psychedelic therapists who paid attention to the concepts of “set and setting” were much different from those who used LSD in unethical contexts (Lee and Shlain, 1985; Dyck, 2008; Barber, 2018; Oram, 2018; Hartogsohn, 2020).

In this paper, I complicate these neat divisions between “pioneers” and “mad scientists” by looking at LSD’s history as a *correctional tool*. While the case of Timothy Leary’s Concord Prison Experiment is well known, a closer look at the history of LSD reveals that the drug’s use in correctional facilities was actually quite widespread in North America and Europe during the postwar period. Drawing on examples from Canada, I show how enthusiasm for LSD’s ability to produce “rapid personality change” (Unger, 1963, p. 119) led many psychedelic pioneers to consider its use in diverse institutional contexts, which often involved vulnerable populations. In correctional settings, this experimentation was usually encouraged by prison reform movements that emphasized the goal of rehabilitation over mere custodial care. As a means of effectively and quickly reshaping personality, LSD therapy fit in with this goal.

## 2. LSD as a correctional tool in 1960s Canada

### 2.1. Duncan Blewett and Nicholas Chwelos at the Regina prison in Saskatchewan

The most often cited example of carefully done LSD therapy during the 1950s took place in Saskatchewan, Canada, where a group of psychiatrists developed the “psychedelic” method. Building on the concept of ‘psychedelic’ that originated with Humphry Osmond, several researchers in the province developed a sophisticated and highly methodical way of using LSD and similar compounds to help their patients have profound and transformative experiences of self-acceptance (Dyck, 2008). This approach was explained in detail in a 1959 handbook written by Duncan Blewett and Nicholas Chwelos, who both worked for Saskatchewan’s Department of Psychiatric Research.

The handbook outlined the kinds of reactions that LSD produced and provided instructions on how therapists could guide patients toward the most therapeutically useful ones. The idea was to help patients achieve “psychedelic reactions,” which provided insight about underlying attitudes and values. But if the drug experience was met with resistance, patients would fall into “escape reactions” or “psychotomimetic reactions,” and not gain any benefit. To increase the chances of psychedelic experiences, Blewett and Chwelos explained that patients needed to be carefully prepared for the experience beforehand and develop a close relationship with the therapist. They also stressed that LSD sessions should be conducted in a comfortable setting and highlighted the value of using various props to guide the experience, such as music, paintings, and flowers (Blewett and Chwelos, 1959).

With this approach, the Saskatchewan group found significant success in patients struggling with alcohol use. By the early 1960s, the results were so promising that the provincial government endorsed psychedelic therapy as a new and effective treatment for alcoholism. The method, as detailed in Blewett and Chwelos’ handbook, became influential among those working with LSD and spread down the west coast and throughout the US.

In a 1958 report to the provincial government, Blewett explained that such success “warrants the use of LSD on a much wider scale.” (Blewett, 1958, p. 1). At this time, he and Chwelos were already working on a pilot study that examined the effectiveness of psychedelic therapy for a group of young, repeat offenders at the local prison in the city of Regina. Roughly 30 males, ranging from adolescence to 23 years old, were involved in the study, and their experiences were documented through a questionnaire following the sessions. As Blewett explained in the report, the young offenders were not responding as positively as other subjects. While about 75 percent did seem to have a psychedelic reaction and reported that they felt more affection and trust toward others during the experience, many also attempted to “fight off the drug effects” and “appeared to be much more confused, tense and suspicious” than other groups (Blewett, 1958, p. 41–47). In short, Blewett felt that the psychedelic method “offers less to prisoners.” (Blewett, 1958, p. 43).

But this lack of response, he suggested, was largely due to the environment in which the inmates lived and the lack of resources to effectively carry out the study. Blewett noted that the living situation at the prison was not conducive to maintaining the insights found while under LSD. If inmates did come to reassess their values and attitudes during the sessions, they returned afterwards to a social situation in which the “values are almost diametrically opposed to those…encountered in the LSD experience” (Blewett, 1958, p. 55). This issue was compounded by the limitations imposed on the study by insufficient time and staff. Ideally, Blewett pointed out, individuals would have several hours of preparatory interviews before and after a psychedelic session and receive as many sessions as needed to achieve progress. However, such measures had “not been feasible” in the pilot study at the prison (Blewett, 1958, p. 56).

With these problems in mind, Blewett suggested that a “thorough reorganization of procedures” was necessary to improve the use of the psychedelic method in the prison context. This required dedicating more effort to preparing those involved in the program for the experience, as well as working with them afterwards. In this way, therapists could help “re-inforce the positive aspects of the experience through follow-up.” In addition, Blewett stressed that the number of psychedelic sessions should be tailored to the needs of each individual, and not determined in advance by the study protocol (Blewett, 1958, p. 60).

### 2.2. Mark Eveson, Florence Nichols, and the Kingston Prison for Women

Another pilot study that assessed the value of using LSD in a correctional context was conducted by the psychologist Mark Eveson at the Kingston Prison for Women in Ontario. As part of a new focus on rehabilitation, Eveson was hired as the prison’s first Chief Psychologist in 1961, following his completion of a postgraduate degree in psychology and psychiatry at the nearby Queens University. At this time, the majority of inmates at the prison were serving sentences relating to narcotic addiction. As a result, Eveson’s primary focus was on developing new methods and facilities to address this problem. However, the prison was overcrowded, with about 110 inmates, and the treatment staff resources were limited to him, along with a social worker and a part time psychiatrist. Eveson recognized that many other prisons were in similar circumstances and emphasized the need to “seek newer and more economic forms of treatment” (Eveson, 1963, p. 25). LSD therapy, he proposed, presented the “possibility of ending criminal involvement” in “approximately eight hours,” a fact that should “arouse intense interest in all concerned in rehabilitation.” (Eveson, 1963, p. 27).

Eveson began the pilot study in early 1961 and ended up giving LSD to 23 women, some of whom were there for addiction related charges. Although he had some familiarity with the drug, he recruited a psychiatrist who was experienced in its therapeutic use to teach the prison staff how to work with it effectively. This psychiatrist was Florence Nichols, a Canadian missionary who had several years of experience with LSD (Gilmore and Somerville, 1998, p. 122). Nichols had just returned from England, where she learned about LSD therapy through Frank Lake, a fellow medical missionary who began using the drug to help patients relive early infancy and birth. Lake encountered the drug while working under the British psychiatrist and LSD therapy pioneer, Ronald Sandison (Lake, 1966, p. xix). After six months of refining her therapeutic technique in England, Nichols returned home to Canada where she started her own practice and gained familiarity with the psychedelic approach developed in Saskatchewan (Barber, 2018, p. 126–127).

The first inmate from the prison to receive LSD was Christine Bauman, a 30-year-old who was serving a five-year prison sentence for fraud. Like most others in the study, Bauman was taken to a nearby psychotherapy center for the LSD session. Nichols gave her a total of 450 micrograms of LSD in one day, followed by an injection of Ritalin to stimulate the experience. During the session, Nichols used lights, music, and various objects to direct the drug reaction. Bauman later reported that the experience was terrible, and that it negatively impacted her personality (Gormerly, 1968). Nevertheless, Eveson and the staff continued with 22 other inmates and planned a larger study.

Eveson had published a brief description of this work in 1963, but the study did not generate controversy until the 1990s, when one of the inmates who was given LSD, Dorothy Proctor, submitted a complaint of mistreatment to Correctional Services Canada. Proctor claimed that she had not consented to participating in the study and that she was given LSD on one occasion in solitary confinement at the prison (Gilmore and Somerville, 1998, p. 11; Proctor and Rosen, 1994). The federal government sponsored an investigation into her allegations, and Proctor launched a lawsuit against Eveson and the Canadian government that was later settled out of court.

### 2.3. Gary Maier and the social therapy unit at Oakridge

A final example of LSD’s use as a correctional tool took place at the Oakridge maximum-security psychiatric hospital in Penetanguishene, Ontario. From the late 1960s to the mid-1970s, psychiatrists at Oakridge used LSD to treat men who had been convicted of crimes relating to violent behavior or sexual abuse.

Oakridge was built in the 1930s as a custodial facility for men labeled “criminally-insane.” These men, who were later diagnosed as “psychopathic” or “schizophrenic,” typically remained at Oakridge for life, and by the 1960s roughly 250 of them lived there. In 1965, in an attempt to move Oakridge beyond mere custodial care, the superintendent launched a therapeutic program that focused on rehabilitating inmates and integrating them back into society (Nielsen, 2000, p. 165). Oakridge’s “Social Therapy Unit” was at the center of these efforts. Established by the psychiatrist Elliot Barker, the Social Therapy Unit used intensive encounter therapy to break down psychopathic defenses and help inmates make meaningful connections with others. One technique to facilitate this aim was to use what Barker referred to as “defense-disrupting drugs”: scopolamine, sodium amytal, and LSD (Nielsen, 2000, p. 182–183). The program intensified in 1967 when Barker introduced “The Total Encounter Capsule,” an 8 × 10 steel room in which groups of inmates stayed for days on end. On some occasions, to promote “genuine encounter between persons,” the inmates would sit in the capsule while naked or after taking LSD (Barker and McLaughlin, 1977, p. 355).

In 1972, the Canadian psychiatrist Gary Maier took over as director of the Social Therapy Unit. Maier had completed a medical degree at the University of Western Ontario several years earlier and had been working at Oakridge as part of his psychiatric residency. While he was enthusiastic about the program that Barker set up, he had a much different perspective on LSD. Whereas Barker understood LSD as a defense-disruptor, Maier used it to “open the door from the inside,” and he shifted the program at the Social Therapy Unit to reflect this perspective (Nielsen, 2000, p. 222–223). Indeed, Maier was well versed in spiritual and countercultural perspectives on psychedelics. He had gone through his own LSD experiences, which gave him an “affirmation of the inner aspect,” and he did some training with the influential psychedelic pioneer, Stanislav Grof (Marshall, 1976).

A year after taking over the Social Therapy Unit, the Canadian government licensed Maier to continue using LSD at Oakridge, and sent him 20,000 micrograms of the drug, the same amount it sent to Barker in 1967 (Maier et al., n.d., p. 4). For the new LSD program, Maier drew on the work of Grof to “facilitate an ‘ego death-rebirth’ experience.” He also focused on developing a therapeutic milieu on the ward to help inmates incorporate the “being values” (eg Maslow’s values of truth, wholeness, beauty) that they experienced while under LSD (Maier et al., n.d., p. 5). Inmates over the age of 18 were “permitted to volunteer” for the program. Those who became involved were put into pairs and then underwent eight weeks of preparation before taking LSD, which included learning about the drug’s effects and the “experimental nature” of the therapeutic approach. In addition, to create a beneficial “head space” for the experience, Maier provided the pairs with a reading list about psychedelics, and they spent “approximately five hours a day” discussing works by authors such as Carlos Castaneda, John C. Lilly, Aldous Huxley, and Timothy Leary (Maier et al., n.d., p. 8–10).

After this preparation, the LSD sessions took place in the Total Encounter Capsule along with Maier and the other member of the pair. Before the session started, Maier used relaxation techniques and “group chanting” to encourage an “atmosphere of trust and warmth” (Maier et al., n.d., p. 15). Experiences tended to fluctuate between periods of relaxation and agitation. After several hours, Maier left the Capsule, but the other member remained “as the main support for the tripper.” (Maier et al., n.d., p. 16). By the end of 1975, Maier had guided 67 LSD experiences in this way. While the psychological metrics did not suggest any clear results, Maier and the superintendent of Oakridge felt that the program was successful. In 1977, a government sub-committee conducted an evaluation of Oakridge and declared that “psychopaths are being treated with success” (Nielsen, 2000, p. 223–225). Experienced LSD therapists also endorsed the program. In 1980, Grof, for example, referred to Maier’s work as an “interesting attempt… to integrate LSD therapy into a complex therapeutic regime under the conditions of maximum security” (Grof, 1980, p. 244).

Yet despite the enthusiasm from these experts, the Oakridge experiment remains one of the most morally shocking examples of LSD therapy. In 2001, the inmates involved in this program sued Barker, Maier, and the Canadian government for inhumane treatment. In 2021 they won their case (Philips, 2022).

## 3. Discussion

As these examples show, interest in LSD as a correctional tool was quite widespread, even among knowledgeable and experienced psychedelic therapists. With excitement about LSD’s potential to efficiently transform personality on a large scale, these therapists introduced psychedelics to diverse institutional settings in hopes that they would serve as effective rehabilitation tools. To many pioneers of psychedelic therapy, prisons seemed like ideal spaces to demonstrate the value that this kind of therapy had for society.

The history of LSD’s use in correctional contexts reveals how the broader institutional matrix of modern industrial societies contributed to the “collective set and setting” within which psychedelics were understood, experienced, and implemented. By conducting psychedelic therapy within prisons, researchers aligned the aims of this therapeutic modality with those of correctional facilities: that is, with the creation of a certain kind of productive and functional citizen. In this way, psychedelic therapy was tied to the biopolitical purpose of these institutions. Scholars have shown that the “psy-disciplines” operate within a political and institutional context that influences their practices as they contribute to the goal of shaping the modern subject (Rose, 1996). As a technique originating from within the psy-disciplines and deployed in institutional settings, psychedelic therapy worked towards this goal. By highlighting this, humanities scholars can better understand why so many therapists were motivated to use psychedelics on vulnerable populations.

Today, of course, psychedelic therapy continues to operate within an institutional matrix that is in many respects similar to that of the postwar era. As advocates and researchers work to medicalize and mainstream psychedelics, they bring them in line with the aims of neo-liberal democracies (Gearen and Devenot, 2021). But from the perspective of current researchers, there are significant differences between now and the 1960s, since today ethical guidelines, Institutional Review Boards, and rigorous approval processes function to protect against unethical uses of psychedelics. From this perspective, examples of controversial psychedelic research in the past were part of an era that lacked these safeguards. However, tendencies exist within the current “psychedelic renaissance” that could lead to the use of psychedelic therapy on vulnerable populations once again. The ever-expanding list of diagnostic indications, enthusiasm about the potential of psychedelic therapy, and the commodification of psychedelics might once again create a situation in which this form of therapy is considered in diverse institutional settings. Consequentially, those working with psychedelics today should keep LSD’s history as a correctional tool in mind when championing the use of these drugs.

## Data availability statement

The original contributions presented in the study are included in the article/supplementary material, further inquiries can be directed to the corresponding author.

## Author contributions

The author confirms sole responsibility for the entirety of the article, including study conception and design, data collection, analysis and interpretation of results, and manuscript preparation.

## Funding

This work was funded in part by a Joseph-Armand Bombardier Canada Graduate Scholarship from the Social Sciences and Humanities Research Council.

## Conflict of interest

The author declares that the research was conducted in the absence of any commercial or financial relationships that could be construed as a potential conflict of interest.

## Publisher’s note

All claims expressed in this article are solely those of the authors and do not necessarily represent those of their affiliated organizations, or those of the publisher, the editors and the reviewers. Any product that may be evaluated in this article, or claim that may be made by its manufacturer, is not guaranteed or endorsed by the publisher.
